# A novel early precursor cell population from rat bone marrow promotes angiogenesis *in vitro*

**DOI:** 10.1186/1471-2121-15-12

**Published:** 2014-03-26

**Authors:** Andreas Brandl, Quan Yuan, Anja M Boos, Justus P Beier, Andreas Arkudas, Ulrich Kneser, Raymund E Horch, Oliver Bleiziffer

**Affiliations:** 1 Department of Plastic and Hand Surgery, Hospital of Erlangen, Friedrich-Alexander-University of Erlangen-Nuremberg, Krankenhausstrasse 12, Erlangen 91054, Germany

**Keywords:** Angiogenesis, Endothelial progenitor cells (EPC), Tissue engineering, Bone marrow, Rat

## Abstract

**Background:**

Some studies demonstrated therapeutic angiogenesis attributable to the effects of endothelial progenitor cells (EPC), others have reported disappointing results. This may be due to the fact that EPC populations used in these contradictory studies were selected and defined by highly variable and differing experimental protocols. Indeed, the isolation and reliable characterization of *ex vivo* differentiated EPC raises considerable problems due to the fact there is no biomarker currently available to specifically identify EPC exclusively. On the other hand traditional differentiation of primary immature bone marrow cells towards the endothelial lineage is a time-consuming process of up to 5 weeks. To circumvent these shortcomings, we herein describe a facile method to isolate and enrich a primary cell population from rat bone marrow, combining differential attachment methodology with cell sorting technology.

**Results:**

The combination of these techniques enabled us to obtain a pure population of early endothelial precursor cells that show homogenous upregulation of CD31 and VEGF-R2 and that are positive for CD146. These cells exhibited typical sprouting on Matrigel™. Additionally, this population displayed endothelial tube formation when resuspended in Matrigel™ as well as in fibrin glue, demonstrating its functional angiogenic capacity. Moreover, these cells stained positive for DiI-ac-LDL and FITC-UEA, two markers that are commonly considered to stain differentiating EPCs. Based upon these observations in this study we describe a novel and time-saving method for obtaining a pure endothelial precursor cell population as early as 2–3 weeks post isolation that exhibits endothelial abilities *in vitro* and which still might have retained its early endothelial lineage properties.

**Conclusion:**

The rapid isolation and the high angiogenic potential of these syngeneic cells might facilitate and accelerate the pre-vascularization of transplanted tissues and organs also in a human setting in the future.

## Background

The term angiogenesis defines a complex physiological process where new blood vessels are formed from pre-existing vessels. In a distinctly different process of vasculogenesis, on the other hand, angioblasts differentiate into endothelial cells *in situ*, assembling into a vascular labyrinth. It is generally believed that EPC derive from a common hematopoietic precursor and contribute to vasculogenesis [1]. Indeed, their vasculogenic and angiogenic potential has been demonstrated in a multitude of experimental and clinical studies of tissue ischemia, thereby making them potentially useful for regenerative medicine applications where generation of new blood vessels is needed. Bioengineered tissues have become an essential element in regenerative medicine and tissue engineering applications [2-4]. Once they exceed a critical size, however, their oxygenation becomes challenging and can only be maintained by a three-dimensional functional vascular network. A variety of experimental strategies have been applied to overcome this problem such as the incorporation of pro-angiogenic growth factors [5,6] or endothelial progenitor cells [7] in these bioengineered constructs. Addition of EPC constitutes a relatively new approach to enhance blood vessel formation in bioartificial tissues [8]. Significant research efforts have focused on isolation, definition, selection and characterization of EPC populations. A precise definition and delineation of precursor cells with potent angiogenic properties is still a controversial subject. It has recently been recommended to avoid the term EPC altogether in favor of a precise definition of cellular phenotype [9] which again is dependent on the respective algorithm and protocol used for cell selection. Given that specific cell surface markers to identify endothelial progenitor cells are unknown, it is impossible to separate the EPC population at an early state of differentiation by using methodology based on magnetic activated cell sorting (MACS). Therefore, other approaches such as the differential attachment method [10-12] have been used to isolate EPC. However, awaiting outgrowth of a pure cell population from the pluripotent heterogenous bone marrow fraction of mononuclear cells (MNC) is very time consuming.

This study introduces the combination of differential cell attachment followed by enrichment of the cells via cell sorting as early as 2 weeks post isolation of the MNC fraction from bone marrow. Our method exploits the ability of endothelial lineage cells to take up increased amounts of acetylated low density lipoprotein (ac-LDL) via specialized scavenger receptors. As a consequence, we were able to enrich a population of putative EPC of rat bone marrow MNC with unusually high purity at an early time point. We characterized their phenotype using Flow Cytometric analyses and their angiogenic potency by tube formation assays *in vitro* in comparison to a rat endothelial cell line.

## Methods

### Animals

Male Lewis rats (Charles River Laboratories, Sulzfeld, Germany) served as donors for the bone marrow. German regulations for the care and use of laboratory animals were observed at all time. The animal care committee of the Unviversity of Erlangen and the government of Mittelfranken approved all experiments. The animals were housed in the Franz-Penzoldt-Zentrum in Erlangen and submitted to a 12-h dark/light cycle with free access to standard chow (Altromin, Hamburg, Germany) and water.

### Cells and culture conditions

The rat endothelium cell line EC52 was used as a positive control for functional experiments, (A kind gift from Prof. Dr. U. Rauen, Institute of Physiological Chemistry, University of Duisburg-Essen). EC52 cells were cultured in RPMI 1640 medium, supplemented with 20% FBS, 4 mM L-glutamine, dexamethasone (720 ng/ml; Roche Diagnostics), penicillin (100 U/ml), and streptomycin (100 mg/ml) in a humidified atmosphere of 5% CO_2_ in air (according to provider’s instructions). Cells were split at ~90% confluency to maintain a constant cell density.

### Isolation of mononuclear cells from rat bone marrow

Bones (femur and tibia of hind legs) from 6-week-old male Lewis rats were repeatedly flushed with PBS containing 2% FBS. The washing fluid was centrifuged and the remaining pellet was resuspended in 10 ml pre-warmed EGM MV2 medium with FBS, VEGF, R^3^-IGF-1, rhEGF, rhbFGF, ascorbic acid and hydrocortisone (PromoCell GmbH, Heidelberg, Germany). This suspension was filtered into a single-cell suspension with a 70-μm Cell Strainer (BD Falcon™, Heidelberg, Germany). Single-cell suspension was carefully underlaid with 5 ml of Histopaque®-1077 (Sigma-Aldrich Chemie GmbH, Steinheim, Germany). The mixture was then centrifuged at 2,000 rpm for 20 min at 20°C without brake to separate the cells into three layers. The white and cloudy interphase which consists of the MNC was gently removed and washed with 10 ml of pre-warmed medium. The pellet was resuspended in complete medium and seeded in 12-well plates with a density of 2x10^6^ cells/well. After 24 h the non-adherent cell population was transferred to gelatin-coated (1%) plates to remove rapidly adherent hematopoietic cells. Only the cell population which was non-adherent after 24 h was subjected to additional evaluation.

### Flow cytometry (FACS) analyses

Cells were stained for the presence of CD31 (AbD Serotec, Düsseldorf, Germany) to demonstrate the presence of endothelial cells, and CD146 (R&D Systems GmbH, Wiesbaden-Nordenstadt, Germany), a cell adhesion molecule that is currently used as a marker for endothelial cell lineage. Additionally, cells were stained for VEGF receptor-2 (KDR, Abcam, Cambridge, UK). All stainings were carried out according to manufacturer’s protocols. Expression of cell surface markers was measured with a FACS-Calibur running the Cell Quest software (BD Biosciences, San Diego, CA, USA). Raw data were analyzed with the FlowJo software (Tree Star, Inc., Ashland, OR, USA). The primary antibody was omitted in the negative controls. The given percentages in brackets represent the mean of 4 independent isolations and FACS-analyses with respect to the upregulation of indicated cell surface markers and the corresponding standard deviation respectively.

### ac-LDL-Uptake and cell sorting

Cells were incubated with 2.5 μg/ml Alexa Fluor® 488-ac-LDL or DiI-ac-LDL (Life Technologies GmbH, Darmstadt, Germany) for 4 h at 37°C and 5% CO_2_. Cells were subsequently washed twice with PBS and directly analyzed by fluorescence microscopy. Pictures were taken with an Olympus IX81 inverted microscope running the cellSens® imaging software (Olympus, Center Valley, PA, USA). To separate and select the cells that had taken up the ac-LDL, FACS analysis and sorting was carried out using a FACS Aria II SORP (BD Biosciences, San Diego, CA, USA). This machine is part of the Core Unit Cell Sorting and Immunomonitoring Facility Erlangen. Positive and negative cell fractions were collected and seeded again in complete medium (EGM2 MV) for further analysis.

### FITC-UEA binding

After incubation with DiI-ac-LDL, the selected cell population was washed twice with PBS and incubated with 5 μg/ml FITC-UEA (Sigma-Aldrich Chemie GmbH, Steinheim, Germany) at 37°C and 5% CO_2._ After an additional washing step with PBS, cells were analyzed by fluorescence microscopy.

### *In Vitro* capillary tube formation assay using matrigel™ and fibrin

For analysis of capillary tube formation, 75 μl of BD Matrigel™ (Becton Dickinson, Heidelberg, Germany), an extracellular mouse sarcoma matrix (Engelbreth-Holm-Swarm tumor) which is a well-known pro-angiogenic stimulus *in vitro* and *in vivo,* was placed into the wells of a 96-well plate (Falcon, Heidelberg, Germany) and incubated at 37°C for 60 minutes. Cells were harvested and suspensions containing 5x10^4^ cells in 150 μl of EGM MV2 medium were seeded onto the Matrigel™-coated 96-well plates and incubated for 24 h at 37°C and 5% CO2. The same procedure was used when cells were resuspended in Matrigel™, only that 1x10^5^ cells were used under these conditions. After clot formation, 200 μl EGM2 MV was added on top. Additionally, cells (5x10^5^) were resuspended in 500 μl fibrin glue (TISSEEL Kit (Fibrin Sealant), Baxter Healthcare Corporation Westlake Village, CA 91362 USA), consisting of the two components Fibrin (1.5 mg/ml, 300 μl) and Thrombin (0.6 IU/ml, 200 μl) and seeded in 48-well plates. After clot formation, 500 μl EGM2 MV was added on top. Capillary tube formation was observed under an inverted Leica DMIL microscope and photos were taken using the Leica application suite software (Leica Microsystems GmbH, Wetzlar, Germany) after 24 h of incubation.

## Results

### Cell morphology and surface marker expression of isolated rat BM-cells 2 weeks post isolation

**Figure 1:**
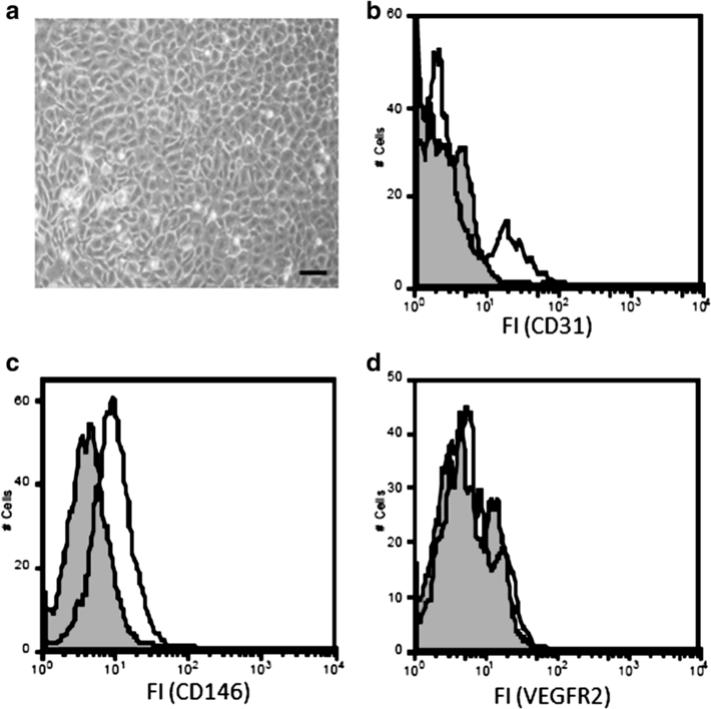
**Flow cytometric analyses 2 weeks after isolation and cultivation of rat bone marrow MNC show heterogeneity of the cell population. (a)** Cells were grown in specialized endothelial cell growth medium (EGM2 MV) for 2 weeks after isolation and their morphology was examined using light microscopy. The scale bar depicts 100 μm. The cells were further analyzed by Flow Cytometry for expression of endothelial cell-specific surface markers including CD31 **(b)**, CD146 **(c)** and VEGF-R2 **(d)**. Grey histograms indicate fluorescence signals of negative controls; white histograms indicate fluorescence signals of specific antigens. Results are representative of 4 separate experiments.

Rat MNC isolated from bone marrow by density gradient and cultured on Gelatin-coated (1%) culture plates in EGM MV2 exhibited distinct cell morphology typical of endothelial lineage cells. These cells formed colonies that showed pronounced “cobblestone” morphology (Figure 1a). Analogous observations were made for the rat liver endothelial cell line EC52 that served as a positive control for functional experiments in this study. Furthermore, 2 weeks post-isolation, the population demonstrated to be composed of heterogenous cells in FACS-analyses. A small fraction of the cells expressed CD31 (Platelet endothelial cell adhesion molecule, PECAM-1) (8.3% ± 2.1) (Figure 1b). CD146, also known as the melanoma cell adhesion molecule (MCAM), was already markedly expressed on the cells with different intensity (15.7 ± 5.1%) (Figure 1c). Cells did not express vascular endothelial growth factor receptor 2 (VEGF-R2/KDR) at this time point (Figure 1d).

### Enrichment and separation of the ac-LDL-positive cell fraction by cell sorting

**Figure 2:**
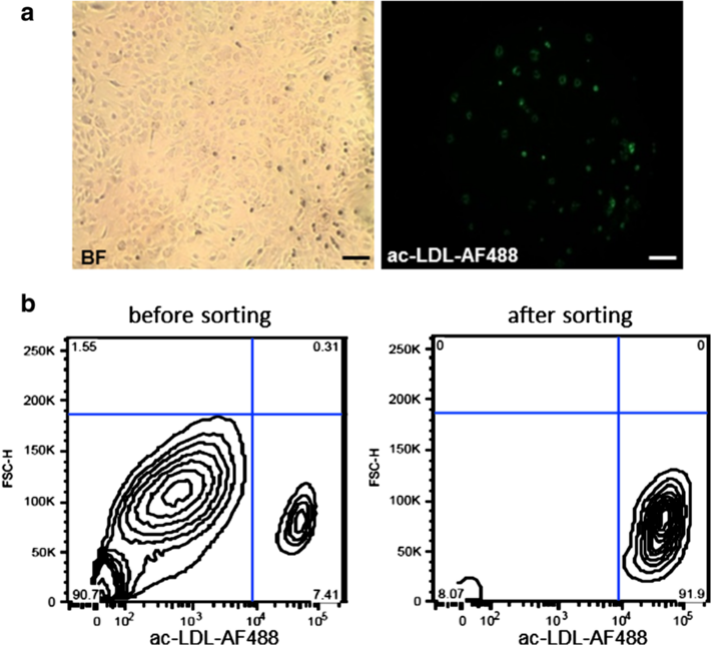
**The heterogenous cell population can be sorted by the ability of cells of the endothelial lineage to take up ac-LDL 488 via specialized scavenger receptors. (a)** Cells were incubated with 2.5 μg/ml ac-LDL-Alexa Fluor® 488 for 4 h at 37°C and 5% CO_2_. Cells were subsequently washed twice with PBS and successful uptake was visualized by fluorescence microscopy (left: brightfield, right: fluorescence). The scale bar depicts 100 μm. **(b)** By using cell sorting technology, cells that have taken up the acetylated LDL-AF488 conjugate were successfully separated from the remaining population with a purity of 92%. Cell distributions are depicted in the plots.

A small population of the differentiated MNC derived from rat bone marrow demonstrated features of endothelial cells, i.e. they incorporated ac-LDL (Figure 2a). This fraction could be separated from the rest of the cell population using cell sorting technology. The cells were enriched and showed a purity of up to 92% (Figure 2b). Both negative and positive cell fractions were collected and reseeded on Gelatin-coated culture plates for further experiments and functional tests.

### *In Vitro* angiogenesis characteristics of sorted ac-LDL-positive and –negative cell fractions

**Figure 3:**
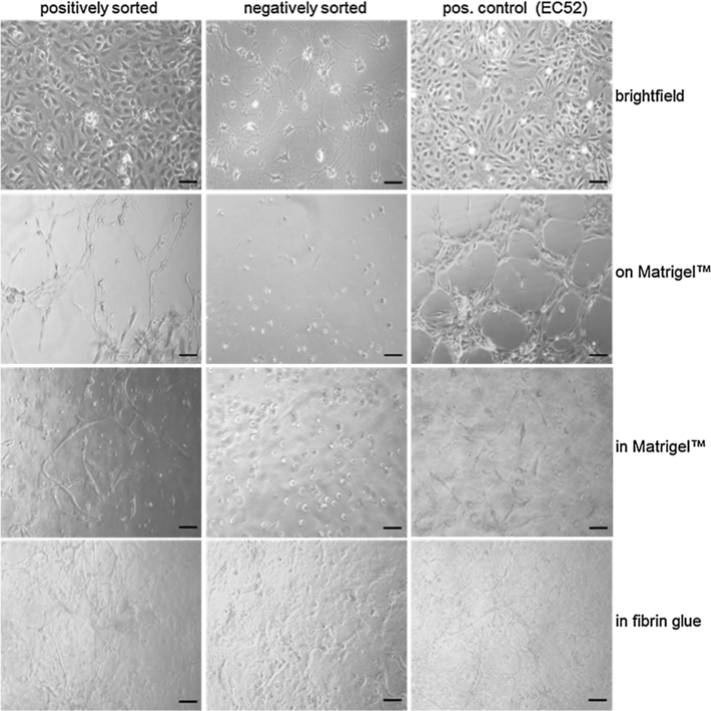
**Angiogenic potence in Matrigel and fibrin glue of positively sorted primary cells is equal to an endothelial cell line.** Positively sorted cells, negatively sorted cells and the liver endothelial cell line EC52 (pos. control) were seeded in cell culture flasks and their phenotype was investigated with light microscopy (top row). 5x10^4^ cells of each population were seeded on Matrigel™ (2^nd^ row) or resuspended in Matrigel™ (3^rd^ row) and 1x10^5^ cells of each population were suspended in fibrin glue (bottom row) to investigate their performance in tube formation assays. Microscope pictures were taken 24 h after cell seeding. The scale bar depicts 100 μm.

Positively and negatively sorted cell fractions significantly differed in terms of morphology. While the positively sorted fraction showed the “cobblestone” morphology, typical of endothelial cells and similar to the endothelial cells serving as positive control, the negatively sorted fraction exhibited an altogether different growth pattern (Figure 3, upper row). Furthermore, the capacity to form tubular networks in 2D as well as in 3D Matrigel™ cultures, an indicator of angiogenic potency, was significantly different when comparing positively- and negatively-sorted cells. Only the positive cell fraction demonstrated network formation on Matrigel™ (Figure 3, 2^nd^ row) and organization into elongated structures that connected to each other after suspension within Matrigel™. After sorting and selection, primary cells appeared to form a higher degree of connected networks than the endothelial cells serving as positive control (Figure 3, 3^rd^ row). Negative control cells were unable to form any networks. Positively-sorted cells were also able to form connected tubular networks in fibrin glue (Figure 3, bottom row).

### Cell morphology and surface marker expression of isolated rat bone marrow-MNC

One week after sorting and reseeding, cells were again analyzed with regard to their morphology and surface marker expression as already described above (Figure 1). Cells still grew in their typical “cobblestone” pattern and the entire population exhibited a uniform appearance with respect to their expression and expected upregulation of CD31 (20.1% ± 6.85%), CD146 (93.25% ± 2.8%) and KDR (26.6% ± 11%) (Figuere 4a-d). Moreover, cells stained positive for DiI-ac-LDL and FITC-UEA (Figuere 4e and f). Cells staining positive for both markers are commonly considered to be differentiating EPC as already reported [13].

**Figure 4:**
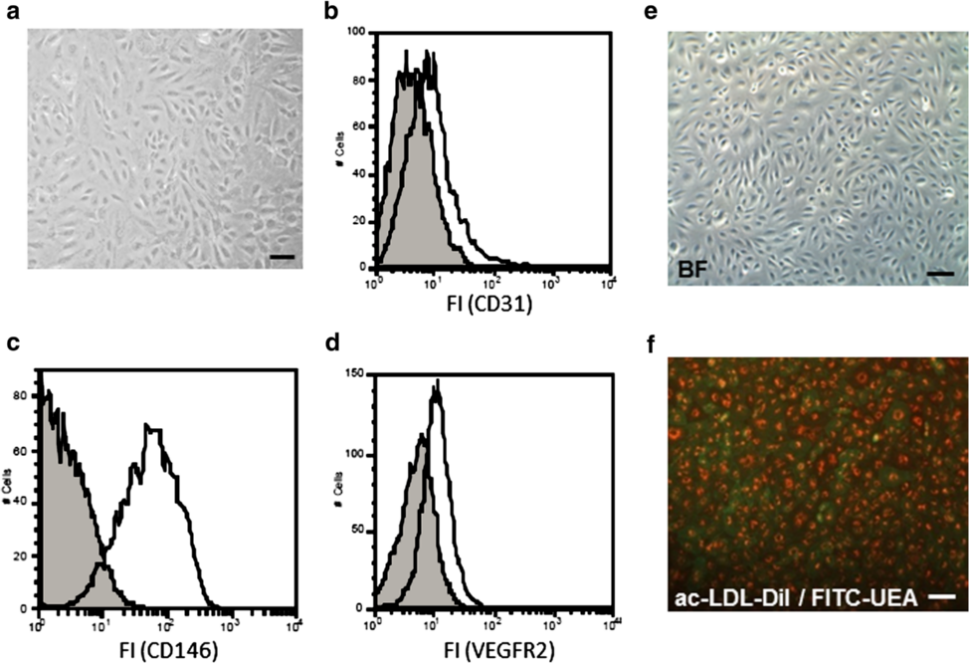
**A homogenous cell population can be detected in FACS-analyses and ac-LDL-uptake assays one week after sorting. (a)** Cells were grown in EGM2 MV for 1 week after sorting and analyzed with a light microscope for their phenotypic appearance. The cells were further analyzed by Flow Cytometry for expression of endothelial cell-specific surface markers CD31 **(b)**, CD146 **(c)** and VEGF-R2 **(d)**. Grey histograms indicate fluorescence signals of negative controls; white histograms indicate fluorescence signals of specific antigens. Results are representative of 4 separate experiments. Cells were incubated with 2.5 μg/ml ac-LDL-DiI for 4 h at 37°C and 5% CO_2_. Cells were subsequently washed twice with PBS and incubated with 5 μg/ml FITC-UEA for 1 h at 37°C and 5% CO_2_. After an additional washing step with PBS cells were analyzed by fluorescent microscopy (**e**: brightfield; **f**: fluorescence overlay). The scale bar depicts 100 μm.

## Discussion

MNC derived from bone marrow constitute a heterogenous cell population and an easy and straight forward method to isolate and select a uniform population of cells with potent angiogenic properties, usually referred to as EPC has not been available to date. Previous studies have focused on magnetic cell sorting [14] and MNC adherent culture [15] in order to separate the EPC. Since there is no unique cell surface marker that clearly defines the properties of EPC, a population separated via magnetic sorting does not necessarily reflect progenitor cells. EPC separated by adherent culture comprise many subpopulations and obtaining a uniform cell population that expresses a homogenous set of endothelial surface markers and endothelial properties is very time consuming. In the past, early and late outgrowth EPC have been distinguished and characterized [10], but the isolation methods are fundamentally based upon their morphology, time of appearance in culture and lifespan and not upon cellular markers or properties.

Our study combines for the first time the benefits of the differential attachment method and the cell sorting technique, allowing for isolation and enrichment of a putative EPC population as early as 2 weeks post-isolation. We observed that MNC from rat bone marrow were cobblestone-shaped and appeared uniform throughout the culture 2 weeks post-isolation. Nevertheless, FACS-analyses of different endothelial cell surface markers exhibited a very heterogenous population. Hence, morphology and appearance of outgrowing cells from a miscellaneous bone marrow fraction should not be considered a uniform population. This finding precludes the application of this early cell population in its entirety for experimental studies and therapeutic use where a precisely defined and uniform population is required.

The existing diversity of the population was emphasized when ac-LDL-uptake experiments were performed. In the past, ac-LDL has been frequently used to confirm endothelial origin of cells based on endothelial-specific increase of ac-LDL uptake [12,16,17]. We exploited the scavenger cell pathway of LDL metabolism to label the cells of the endothelial lineage within the heterogenous bone marrow population and separated the positive from the negative fraction using the cell sorting technique as early as 2 weeks post-isolation, thereby generating a cell population with a very high purity rate of 92%.

The capillary tube formation assay using extracellular matrices such as Matrigel™ has been a standard *in vitro* assay [8,11,18] to demonstrate the angiogenic potency of endothelial cells. In our study, the positively sorted cell fraction demonstrated capacity to form tubular structures and networks on and within Matrigel™ just as efficiently as the control endothelial cell line EC52, while the negatively sorted cells failed to do so. This clearly emphasizes that it is highly important to separate the cells of this heterogenous early population before therapeutic use. Additionally, the positively sorted cell fraction showed network formation when the cells were suspended in fibrin glue. Fibrin is a versatile biopolymer and the prototypical extracellular matrix for tissue regeneration and wound healing applications not only in animal studies but even more so for clinical use where Matrigel™ cannot be employed given that it is of tumor origin. Fibrin, on the other hand, is biocompatible, biodegradable, easily processable and, most importantly, approved for clinical use. It has also been used in a wide variety of experimental studies towards generation of vascularized bioartificial tissues as an extracellular matrix, cell carrier and growth factor release system [5,19,20].

Our positively sorted cell population showed up-regulation and expression of CD31 and KDR with different intensity. These molecules are frequently used as markers to demonstrate the presence of endothelial cells [11,12,21,22]. All cells stained positively for CD146, a cell adhesion molecule which is currently used as a marker for endothelial cell lineage [11,23,24]. All markers exhibited a uniform expression throughout the entire cell population. Additionally, all the cells incorporated ac-LDL and bound the lectin UEA-1. As previously reported [13], cells staining positively for both markers are commonly considered to be differentiating EPC. These findings demonstrated homogeneity and uniformity of a cell population with high angiogenic potential after sorting. Taken together, our findings demonstrate that MNC from rat bone marrow show angiogenic characteristics which are commonly associated with EPC, including cobblestone-like morphology, lectin-binding, ac-LDL-uptake, tube formation on and within Matrigel™ or Fibrin and expression of endothelial cell surface markers. In accordance with others [9], we do not refer to this enriched population as EPC, but rather characterize them as early bone marrow-derived cells of endothelial lineage with high angiogenic potential *in vitro*. As a prospect, *in vivo* experiments with this newly isolated cell population have already been carried out by our group and will soon be published and discussed elsewhere. Briefly: We used a syngeneic arteriovenous loop (AV-loop) model in the rat which is a solitary *in vivo* system that is used for the microsurgical vascularization of bioartificial tissues. It illustrates the development of an emerging three-dimensional system of blood vessels and is hallmarked by its high standardization and evaluation properties. We isolated primary endothelial cells from rat bone marrow (this study) and tested their angiogenic potential in the AV-loop model remarkably soon after cell isolation. Our results demonstrate that this primary endothelial cell population effectively contributes to neo-vascularization not only through paracrine effects but also by their incorporation into the newly built vessel walls. The rapid isolation and the high angiogenic potential of these syngeneic cells might facilitate and accelerate the pre-vascularization of transplanted tissues and organs in a human setting in the future.

## Conclusion

Since there is no unique cell surface marker that clearly defines the properties of EPC, it is difficult to separate this fraction at an early time point from a heterogenous cell population isolated from total bone marrow. Our method allows for generation of a defined cell population as early as 2 to 3 weeks after isolation for experimental use in angiogenesis studies *in vitro* and *in vivo* which is characterized by high purity and retains progenitor cell properties. Based upon our findings we define this population as an early bone marrow-derived endothelial cell lineage with high angiogenic potential *in vitro* proved by FACS analyses with regard to upregulation of specific endothelial cell surface markers, the ability of sprouting and tube formation on and in Matrigel™/fibrin glue as well as positive staining for DiI-ac-LDL and FITC-UEA.

## Abbreviations

EPC: Endothelial progenitor cells; MACS: Magnetic activated cell sorting; MNC: Mononuclear cells; ac-LDL: acetylated low density lipoprotein; FACS: Fluorescence activated cell sorting; VEGF-R2: Vascular endothelial cell growth factor receptor 2; KDR: Kinase insert domain receptor.

## Competing interests

The authors declare that there are no conflicts of interest that would prejudice the impartiality of this scientific work.

## Authors’ contributions

AB, OB and UK designed research. AB and QJ performed research. AB, OB, QJ, AMB, AA, JPB, and UK analyzed data. AB and OB wrote the manuscript. All authors read and approved the final manuscript.
